# A Solitary Choroidal Mass with Spontaneous Resolution

**DOI:** 10.1155/2020/8882617

**Published:** 2020-12-10

**Authors:** Fariba Ghassemi, Nazanin Ebrahimiadib, Hamid Riazi-Esfahani, Hassan Khojasteh, Zahra Mahdizad, Elias Khalili Pour

**Affiliations:** Translational Ophthalmology Research Center, Farabi Eye Hospital, Tehran University of Medical Sciences, Tehran, Iran

## Abstract

**Background:**

To report an atypical case of a transient choroidal mass lesion with spontaneous resolution. *Case Presentation*. A solitary choroidal mass with an overlying neurosensory retinal detachment was seen in an otherwise healthy 31-year-old female. General physical examinations and serum chemistry were unremarkable. The patient had spontaneous resolution two weeks after initial examination without treatment.

**Conclusions:**

Inflammatory choroidal masses may be self-limited, but complete diagnostic measures must always be performed in these patients to distinguish between important causes such as tuberculosis, sarcoidosis, and tumors.

## 1. Background

Choroidal masses have wide differential diagnoses, such as tuberculosis, sarcoidosis, inflammatory diseases, and tumors, and timely diagnosis and treatment of each of these diseases are very important. We report an atypical case of a transient choroidal mass lesion that is spontaneously resolved without any treatment.

## 2. Case Presentation

**Figure 1:**
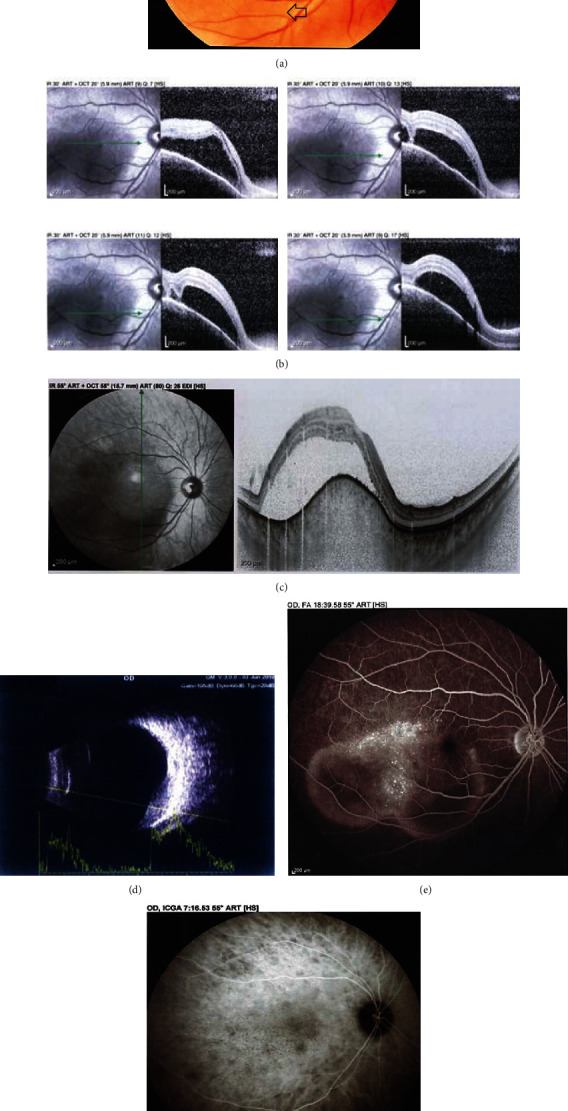
Initial examination of the right eye. Color fundus photo shows subretinal fluid (arrows) and elevated subretinal mass extending beyond the inferior arcade (a). Horizontal scans of optical coherence tomography (OCT) from the fovea to inferior vascular arcade shows subretinal fluid with a dome-shaped elevated choroidal mass in the macular area (b). Vertical scan of enhanced depth imaging optical coherence tomography shows a homogenous hyporeflective elevated lesion in the choroid (c). Ultrasonography shows a hyperechoic choroidal mass with medium to low internal reflectivity (d). Fluorescein angiography in the late phase shows the pooling of dye in the subretinal area and multiple hyperfluorescent dots over the lesion (e). Midphase indocyanine green angiography reveals multiple small, round, hypocyanescent dots in the posterior and midperipheral fundus (f).

A 31-year-old woman was referred to the retina ward of Farabi eye hospital with a history of sudden decreased vision and metamorphopsia in OD for 3 days. On clinical examination, the best-corrected visual acuity was 20/40 and 20/20 in OD and OS, respectively; with +3.00 diopters of hyperopic refraction in OD and Plano refraction in OS. Funduscopy of the OS was normal and the OD was remarkable for a serous retinal detachment (SRD) and a 4 to 5-disc diameter submacular elevated mass with extension beyond the inferior arcade (Figure 1(a)). Anterior segment examination was normal in both eyes without inflammatory findings. No cells or flare in the vitreous cavity, signs of retinitis, or vasculitis were observed.

Spectral-domain optical coherence tomography (SD-OCT) confirmed a dome-shaped elevated choroidal mass with associated SRD (Figure 1(b)) OD. Enhanced depth OCT (EDI-OCT) of the OD demonstrated a homogenous hyporeflective elevated choroidal lesion, with compression of the choroidal vascular structures (Figure 1(c)). Ultrasonography confirmed a hyperechoic choroidal mass (Figure 1(d)). Early-phase fluorescein angiography (FA) showed pooling consistent with SRD and multiple hyperfluorescent dots over the mass with the persistence of these dots through late phases without disc leakage (Figure 1(e)). Indocyanine green angiography (ICGA) revealed diffuse and multiple foci of small, round, hypocyanescent dots in both early and late phases (Figure 1(f)).

**Figure 2:**
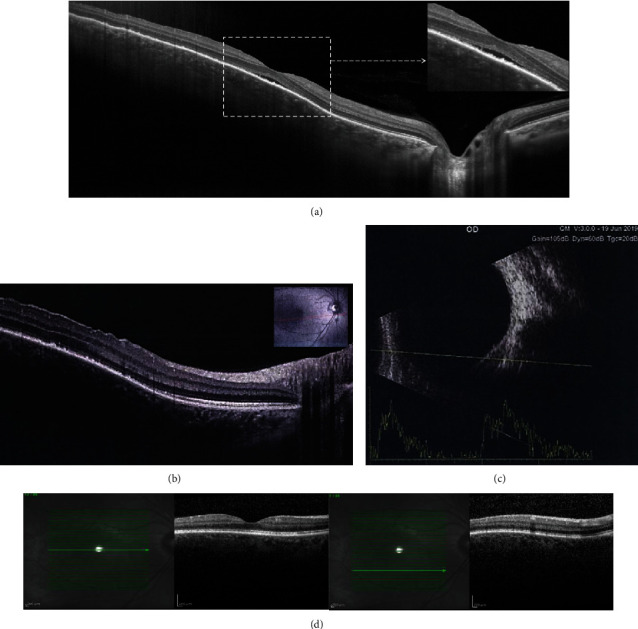
Follow-up examinations. Optical coherence tomography two weeks after initial presentation showed improvement of subretinal fluid, decreasing the size of the choroidal mass, disruption of the ellipsoid zone, and accumulations of hyperreflective material of variable size and shape under the fovea (a) and the corresponding retina over the choroidal mass (b). Ultrasonography two weeks after the initial presentation revealed decreasing the size of choroidal mass (c). OCT one month after initial presentation showed near-complete resolution of choroidal mass and SRF, EZ restoration, and disappearance of hyperreflective materials over RPE and outer retina.

A complete metastatic workup for detecting malignancy including genitourinary system evaluation (Pap smear, ultrasonography, and urine analysis), gastrointestinal system analysis (upper and lower endoscopy, liver function tests, and abdominopelvic computed tomography with contrast), breast examination, and consult with an internist was done, and all were negative for any malignancy. Other evaluations including sarcoidosis (serum angiotensin-converting enzyme and calcium), tuberculosis (chest X-ray, QuantiFERON Gold, and purified protein derivative (PPD) skin test), syphilis (The Venereal Disease Research Laboratory test (VDRL) and rapid plasma reagin (RPR)), and lupus (Anti-nuclear antibodies (ANA), anti-double-stranded DNA antibody (anti-dsDNA)) were negative. Inflammatory markers (erythrocyte sedimentation rate and C-reactive protein) were within normal limits, and the chest X-ray showed no abnormalities. Two weeks after initial evaluation the patient showed spontaneous resolution with improvement in central vision (BCVA: 20/25) and metamorphopsia. Reevaluation was consistent with decreased SRD and choroidal mass volume. Interestingly, there was disruption of the ellipsoid zone (EZ) and multifocal accumulation of hyperreflective material not previously seen on initial OCT (Figures 2(a) and 2(b)). Ultrasonography confirmed the decrease in the size of choroidal mass (Figure 2(c)).

One month after initial presentation visual acuity returned to 20/20 without any refractive error or symptoms. OCT and ultrasonography showed nearly complete resolution choroidal mass effect, SRD, and EZ disruption (Figure 2(d)).

## 3. Discussion and Conclusion

The differential diagnosis of a solitary choroidal mass is broad, including inflammatory, neoplastic, and infectious diseases [1]. Multiple hypofluorescent spots on mid- and late-phase ICGA are indicative of an inflammatory process involving the choroid, such as Vogt-Koyanagi-Harada disease, acute multifocal posterior placoid pigment epitheliopathy (APMPPE), multifocal choroiditis, posterior scleritis, lupus choroidopathy, syphilis, tuberculosis, and sarcoidosis.

Sarcoidosis can affect the eyes in up to 79% of patients with systemic disease, but ocular involvement as a presenting symptom occurs in less than 30% of cases [2]. Although granulomatous or nongranulomatous uveitis is the most common finding, choroidal involvement is a rare manifestation in the absence of anterior uveitis (approximately 5% of patients), and a unifocal lesion is very rare [2, 3].

Kawasaki et al. reported a case presented with a mass resembling ocular metastases in association with Bartonella Hensle that despite treatment ended up with low visual acuity [4]. Our patient had a full recovery and did not show macular star or disc swelling and did not have any history of cat exposure, so that is so unlikely to have Bartonella.

History and laboratory workup, as well as the course of the disease, were not compatible with another diagnosis such as tuberculosis, syphilis, lupus, APMPPE, and multifocal choroiditis.

Even rare, nodular posterior scleritis has been reported [5] and can be considered as another possible diagnosis. Although sudden onset, loss of vision, SRD [5], and findings in FA (a localized starry sky appearance) and ICGA (hypofluorescent dark dots present up to the intermediate phase of angiography) [6] are compatible with the diagnosis but contrary to our case, majority of posterior scleritis patients present with ocular pain (78%) and edema in Tenon space (“T” sign in ultrasonography), and only 13% of patients exhibit subretinal localized granuloma [7–9]. To our knowledge, there is no report of spontaneous resolution of nodular posterior scleritis without any treatment.

MEWDS was first described in 1984 as a transient chorioretinal disease of obscure etiology and regularly influences healthy young ladies. The most prominent feature of the disease is the presence of numerous, tiny yellow-white spots that resolve spontaneously within a few weeks [10, 11].

Full recovery of BCVA and transient course in our case excluded malignancy and narrowed the differential to infectious diseases such as tuberculosis and bartonellosis and inflammatory etiologies such as sarcoidosis, posterior scleritis, and atypical forms of multiple evanescent white dot syndrome (MEWDS). Our patient's fundus examination in the initial presentation did not show the classic white dots of MEWDS. Presumably, the presence of a large amount of SRD could obscure this perception [12–14]. Considering hypofluorescent spots in ICGA, the disruption of the EZ and the accumulation of hyperreflective material in the outer retina may characterize an atypical subtype of MEWDS not previously described.

Shields et al. have previously described 60 patients with an idiopathic inflammatory condition characterized by a solitary choroidal mass without a specific cause and named this condition solitary idiopathic choroiditis (SIC). They made the diagnosis clinically and described the course of disease in two stages: active and inactive. Fifty percent of subjects showed late-phase hypocyanescence over the choroidal lesion. Our case was similar to a typical active SIC, but the case did not show the complete inactive stage characteristics, including discrete, nummular, yellow-white lesion without SRD [15].

In conclusion, inflammatory choroidal masses may undergo spontaneous resolution. A complete diagnostic workup should be performed in these patients to differentiate them from other etiologies.

## Data Availability

The data generated during the present study is available upon request from the corresponding author.
